# The Phononic Properties and Optimization of 2D Multi-Ligament Honeycombs

**DOI:** 10.3390/ma17102369

**Published:** 2024-05-15

**Authors:** Yiguo Yin, Wei Guan, Xing Kou

**Affiliations:** Department of Astronautics and Mechanics, Harbin Institute of Technology, Harbin 150001, China; yinyiguo3256@sina.com (Y.Y.); koux1995@163.com (X.K.)

**Keywords:** ligament structure, band gap, frequency–response, wave propagation

## Abstract

Honeycomb structures have attracted much attention for their excellent characteristics of reducing vibration and noise in recent years. In this study, through band analysis of different ligament structures, we aim to optimize the design of a steel structure that can isolate most of the noise in the 1500–5000 Hz range. The present study examines several different chiral structures. We calculate the band gaps of chiral structures under different geometric configurations and identify the variations in band gaps with geometric layouts. It is found that compared to other chiral structures, the triligaments chiral structure exhibits excellent band gap characteristics. The calculation results demonstrate that enhancing axial symmetry while filling central nodes can effectively enhance the structure’s band gap properties. Frequency–response functions of different lattice structures are computed, and the results align with the calculations of band structures. This study then analyzes the influence of the number of periods on the magnitude of vibration attenuation, revealing that under the same number of periods, the wider the band gap of the structure, the greater the vibration attenuation. Both the triligaments chiral structure and the vertical triligaments structure possess ideal band gap widths, effectively suppressing wave propagation. Subsequently, harmonic response analyses and transient wave calculations further validate the accuracy of the band structure and frequency–response curve calculations. Our study results provide a new way to design a sound insulation structure that can isolate noise signals within the frequency range from 1500 to 5000 Hz in engineering.

## 1. Introduction

Honeycomb structures are widely used in various engineering fields thanks to their outstanding stiffness-to-weight ratio, sound insulation, heat preservation, shock resistance, and so on [1,2,3,4,5]. The performances of honeycombs are strongly dependent on the lattice topology or spatial arrangement. The static behavior of typical honeycomb topologies such as square, triangular, re-entrant hexagonal, Kagome, and chiral has been studied deeply. Various honeycomb structures have different applications in engineering. The resistance and the yield strength of different honeycombs (triangular, square, hexagon, and re-entrant hexagon structures) are studied to restrain vibration and to absorb energy [2,6,7,8,9]. The honeycomb structures of ligaments exhibit auxetic behavior to enhance their mechanical response, improving characteristics such as fracture toughness and resistance to bending and compression [10,11,12,13].

Meanwhile, the acoustic properties of honeycomb structures are also one of the hottest topics of research. The effects of vibrate absorption and noise reduction should be paid more attention. The Honeycomb has the advantage of damping vibration and absorbing energy [14,15,16,17,18,19]. Many periodic honeycomb sound insulation materials are widely used in engineer applications due to the development of technology, like the designing of sound insulation materials for armors of aircrafts and automobiles [20,21]. The periodic honeycomb structures also have a great significance in the application of metamaterials and the development of seismic engineering [22,23,24,25]. In general, the periodicity of the lattice honeycomb structure determines the band in which elastic waves are allowed to propagate or not. The chiral structures are typical periodic honeycombs which are made up of the ring structures connected by the ligaments. By considering chiral structures as combinations of connected frames, numerical models are established to study the effects of cell structure on band gap. Many simulation studies have reported various aspects, including the dispersion characteristics and phase constant surfaces of different structural geometric parameters [26], the vibration band gap characteristics of the homogenous triligaments chiral structure, and the vibration characteristics of innovative re-entrant–chiral elastic metamaterials [27]. Additionally, the wall thickness, the sizes of ribs, and the ring radius are also studied intensively [6,7,28].

Nevertheless, previous studies do not compare the band gaps of different chiral structures of ligaments. The description of parametric modeling is not clear. Moreover, no effective modeling method has been proposed to change the geometry of the structure without changing the size of the unit cell. In this work, we focus on the number of the ligaments and the geometric parameters of the structures. The structural band gaps of different ligament lengths and central node ratios are calculated in the case of keeping the size of the unit cell unchanged. In other terms, the symmetric structures of the ligaments are divided into chiral and axisymmetric structures. We study the differences between them to compare the band gap of the different ligaments and select the best structures for specific analysis. According to the above analyses, our aim is to design a ligament structure to isolate a specific frequency (1500–5000 Hz) in acoustic fields. As we know, steel is readily available in engineering applications. Large-sized steel-plate sound insulation materials operating within the 1500–5000 Hz frequency range are commonly utilized in larger industrial equipment, power plants, ships, trains, airplanes, and other settings requiring enhanced sound insulation performance. These materials have proven effective in reducing noise emissions from larger mechanical equipment, thereby enhancing the comfort and tranquility of the work environment. Large-sized steel-plate sound insulation materials typically exhibit superior sound insulation capabilities and increased durability, rendering them suitable for use in challenging environments [29].

The rest of this paper is organized as follows. Firstly, we briefly verify the correctness of the finite element calculation of the band structure. The chiral structures of three ligaments, four ligaments, and six ligaments are simulated by parametric modeling to obtain a variation rule. We aim to find the lattice structure with the best band gap characteristics. Then, we calculate the frequency–response curve of the lattice structure. On the one hand, the correctness of band structure calculations is verified. On the other hand, the attenuation of the structure on the elastic wave is obtained. By means of harmonic response analysis, simple harmonic loads with frequencies in the band gap and passband are applied to the structure to obtain the displacement response of the structure. The attenuation effect of the structure on the vibration within the band gap can be intuitively seen. Using the idea of robust analysis, we study the influences of manufacturing error on the honeycomb structures. Finally, the transient fluctuations in the symmetric triligaments structures are calculated, respectively, to compare the displacement responses at different times.

## 2. Geometry Parameters and Properties

In this study, we analyze the unit cell of a two-dimensional periodic structure and explore the band gap characteristics of lattice structures using the Bloch–Floquet theory [30]. We examine band diagrams for periodic lattices with varying ligaments and geometric parameters, providing a comprehensive evaluation and discussion of the results. In this study, only propagating waves are computed (i.e., real part of wave number). Moreover, some scholars have also studied the characteristics of evanescent waves (i.e., imaginary part of wave number) for 2D honeycomb structures [31,32]. A viable modeling approach for altering the structural geometry while maintaining the unit cell’s size is presented.

**Figure 1:**
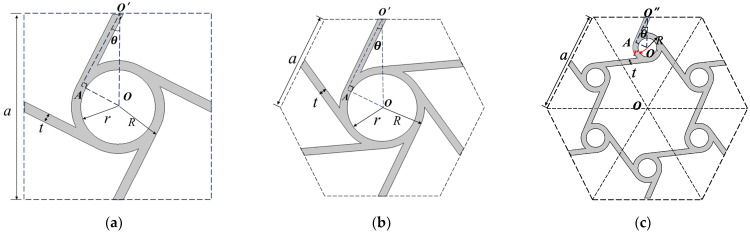
The geometrical structures of chiral structures of different numbers of ligaments. (**a**) Four-ligament chiral structure; (**b**) six-ligament chiral structure; (**c**) triligaments chiral structure.

We use steel materials to design the structures. Figure 1 shows the geometric structures of chiral structures of different numbers of ligaments. For the geometric model, the chiral lattice of four ligaments is a square lattice. Its geometric cell is shown in Figure 1a. The unit cell side length *a* of different ligaments is 1 m. Unless otherwise specified, the unit cell dimensions and thickness of various ligament structures below are the same. We set the outer diameter of the central ring as *R*, the inner diameter as *r*, and the wall thickness and ligament width of the central node as *t*. The ligament is tangential to the central node. $L_{OA}=R-t/2$, $\theta =\sin^{-1}\left(2R-t\right)$, and $O^{\prime }$ is the midpoint of the side.

Figure 1b illustrates the chiral lattice geometric cell with the six ligaments, where the wave vector positioning on the periodic boundary differs from that of the square lattice. The midpoint of each side of the hexagon represents the center of the ligament. Parametric modeling can be established using geometric relations, with the edge length *a* of the unit cell set at 1 m. The ligament is tangent to the central node. Therein, $L_{OA}=R-t/2$, $L_{OO^{\prime }}=\sqrt{3}/2$, and $\theta =∠OO^{\prime }A=\sin^{-1}\left(\left(2R-t\right)/\sqrt{3}\right)$. Similar to the lattice with four ligaments, the center of the ligament is the midpoint of each side of the hexagon.

Figure 1c illustrates the triligaments chiral lattice geometric cell. By connecting the diagonal points with dashed lines, six equilateral triangles are formed. Each triangle includes a central node and three ligaments. The ligaments are rotationally symmetric about the central node, and the structures within these six triangles are rotationally symmetric about the center of the regular hexagon. Point O is center of the regular hexagon. Point O′ is the center of the triangle. Point $O^{\prime \prime }$ is the midpoint of the sides of the regular hexagon. We set the radius as R of the central node, and the ligament width as t. In the triangle, O′O′′A $L_{O\prime A}=R-t/2$ and $L_{OO^{\prime }}=\sqrt{3}/6$. Therein, $\theta =∠O\prime O^{\prime \prime }A=\arcsin\left(\sqrt{3}\left(2R-t\right)\right)$.

## 3. Band Gap Properties of the Chiral Structures

**Figure 2:**
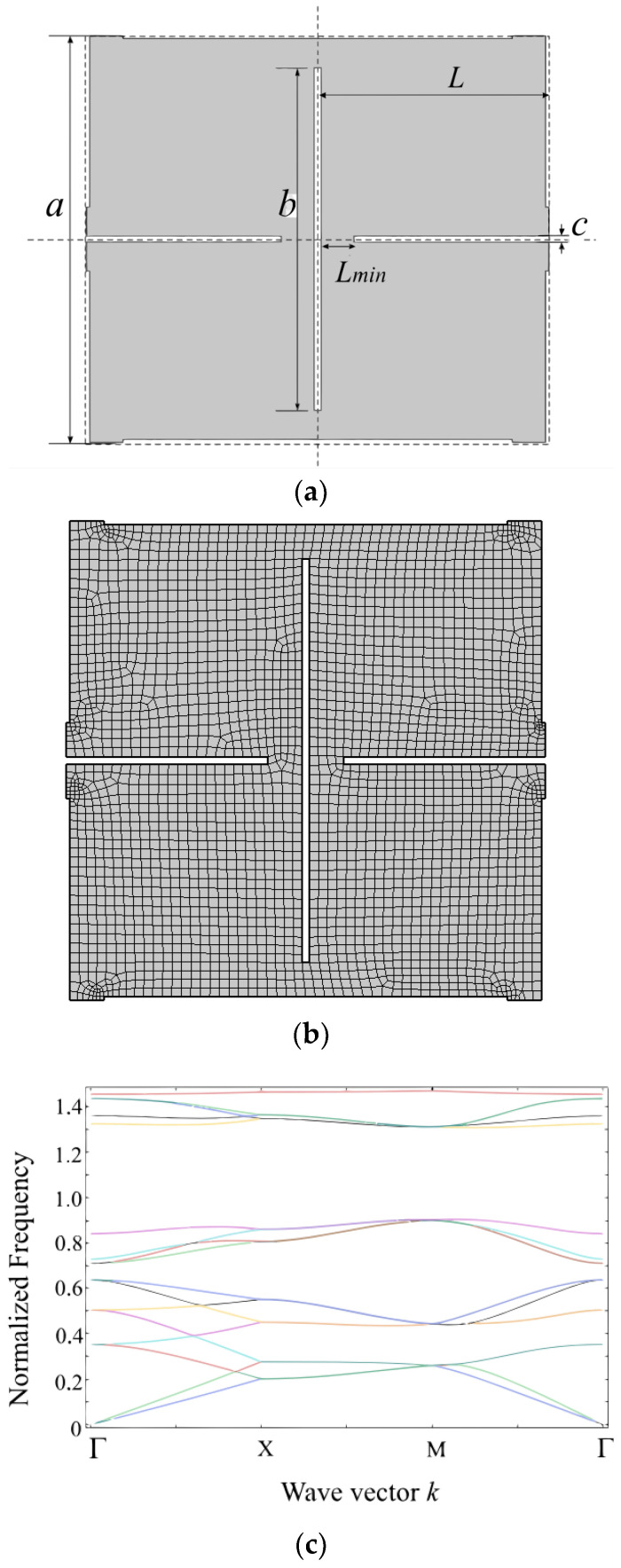
The ultra-low porosity structure model (**a**); (**b**) the structure with meshes; (**c**) the band gap structure.

To verify the accuracy of the finite element method calculations, validation was performed using the ultra-low porosity structure material studied in [33]. This structure consists of rectangular holes intersecting each other on a flat plate, with the unit cell structure shown in Figure 2a. Figure 2b shows the mesh of the structure. We use a quadrilateral grid to divide the model. And the number of the finite elements are 2703. The band structure is shown in Figure 2b. We present the band diagram using COMSOL5.6, with identical porosity values, ψ = *πab*/*L*^2^ = 0.05 and *a/b* = 52.7, where ψ represents the aspect ratio of rectangle pores, *L* is the center-to-center distance between adjacent pores, and *a* and *b* are the major and minor semiaxes of each pore, respectively. The material’s Young’s modulus *E* is 1750 MPa, the material’s density ρ is 1174 kg/m^3^, and the Poisson’s ratio *ν* is 0.35. When the porosity of the unit cell structure is set to *φ, a, b*, and *c*, finite element analysis is carried out. The calculation results are expressed in reduced frequency *f_c_ = f*⋅*a/c*, where a is the lattice constant and *c* is the shear wave velocity. By comparing the result presented in Figure 1c with the literature [34], it can be observed that the trends of characteristic frequencies in the band structure are consistent, and the widths of the band gaps perfectly match with them. Letters Γ, X, and M in Figure 2b represent the high symmetric point of the irreducible Brillouin region, which can be found thoroughly explained in reference [35]. Different color lines indicate different dispersion relationships. The result demonstrates the accuracy of calculating band structures using the finite element method.

### 3.1. Band Gap Properties with the Different Radius R of the Center Node

**Figure 3:**
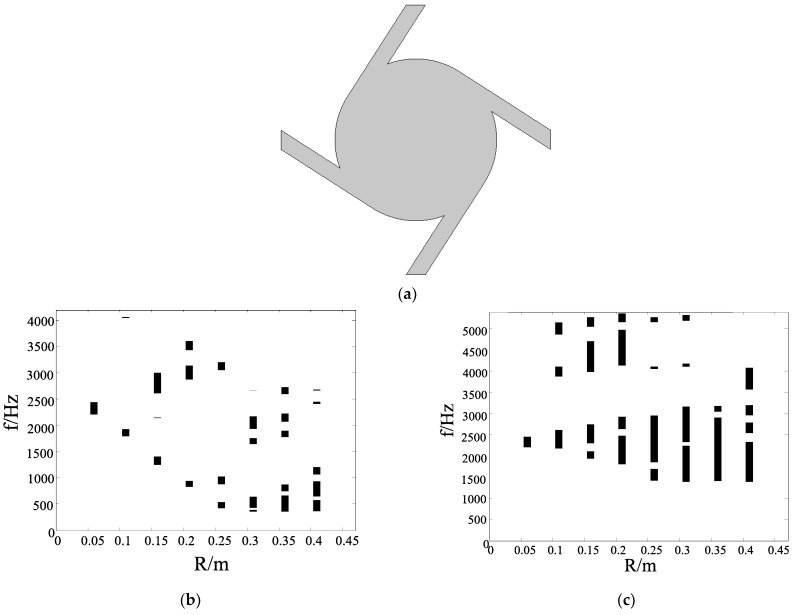
Band gap properties of the four-ligament structure. (**a**) A solid structure; (**b**) a hollow chiral structure; (**c**) a solid chiral structure.

In this subsection, we discuss the band gap properties of the chiral structures. Considering the rationality of the structure, the *R* of four ligaments is not greater than 0.5 m in theory. The band gap of each structure is calculated from 0.05 m to 0.4 m, with 0.05 m as the step length when *t* = 0.05 m. The changing *R* can reflect the ratio of the ligament length *L* and the distance of adjacent nodes with the unchanged unit cell size. We change the radius of the central node *R* with *t* = 0.05 m. Figure 3 shows the band gap properties of hollow and solid four-ligament structure. A hollow four-ligament structure is shown in Figure 1a. A solid four-ligament structure is shown in Figure 3a. Unless otherwise specified, hollow and solid structures of different numbers of ligaments are similar below. As can be seen from Figure 3b, when the size of the central node is gradually increased, the band gap of the structure gradually moves from high frequency to low frequency. After reaching a certain value, the initial frequency of the first band gap does not change much. In addition, it can be seen from the band structure diagram that the position of the first band gap appears between low-order characteristic frequencies. The total width of the band gap gradually increases, which is due to the increase in the radius and the relative decrease in ligament length. It can be found that the band gap is scattered and not ideal. The “scattered band gap” describes a narrow range of band gaps. The spacing between the band gaps is wide, giving it a scattered appearance rather than a centralized one.

In Figure 3c, when the central node becomes solid, we can find that the starting frequency of the first band gap shifts toward the lower frequency as the radius *R* increases. After the radius reaches 0.25 m, the starting frequency of the band gap does not decrease further when the radius continues to increase. However, it can be found that the starting frequency of the first band gap has changed from about 360 Hz to 1400 Hz. This is mainly due to the Bragg scattering mechanism, where the wavelength of the elastic wave corresponding to the center frequency of the first band gap is about twice the lattice constant *a*, which is consistent in this case. The hollow chiral structure has no filling inside the central node, and the out-of-plane stiffness of the chiral structure is lower compared to when the central node is solid.

**Figure 4:**
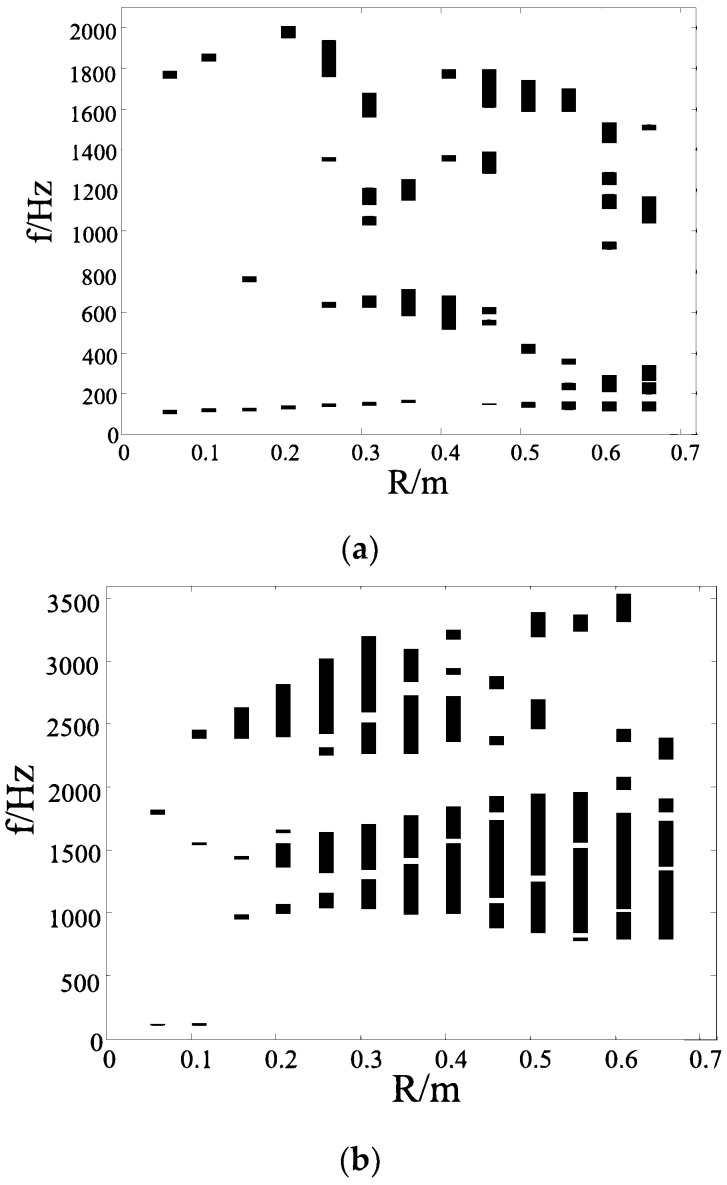
Band gap properties of the six-ligament structure. (**a**) A hollow chiral structure; (**b**) a solid chiral structure.

Figure 4 shows the band gap properties of the six-ligament structures. In Figure 4a, considering the rationality of the structure, the width *t* of the ligament is calculated from 0.05 m to 0.66 m, with 0.05 m as the step length based on the condition of *t* = 0.05 m. When the central node is hollow, the structures are similar to that of four ligaments. After becoming solid structures, the band gap distributions are relatively centralized. It can be seen in Figure 4b that the band gap width of the structure gradually increases by increasing the radius of the central node, except for when the radius of the central node is small, the band gap of the structure is small and not very satisfactory. The band gap widths of the structure gradually rise with the increasing radius of the central node.

**Figure 5:**
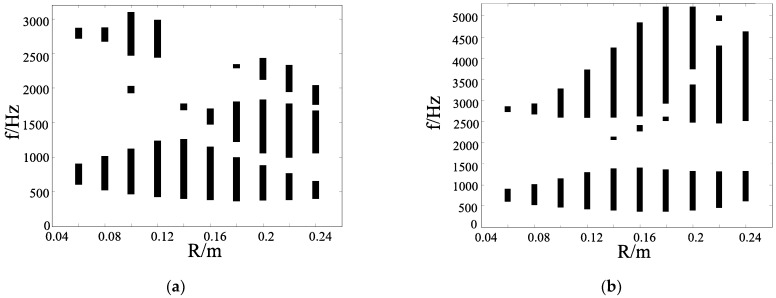
Band gap properties of triligaments structure. (**a**) A hollow chiral structure of three ligaments; (**b**) a solid chiral structure of three ligaments.

Figure 5 shows the band gap properties of the triligaments structures. In Figure 5a, when the central node of the three-ligament chiral structure is hollow, the band gap characteristics of each structure are already good, and it can be seen that the band gap of the structure exhibits certain patterns. For example, the width of the first band gap increases and then decreases as the radius of the central node increases. When the radius reaches nearly 0.14 m, the width of the first band gap reaches its maximum. It can be seen that the total width of the band gap tends to increase with the increase in the radius of the central node later. The band gap characteristics are better compared to other chiral structures.

In Figure 5b, when the center node is becoming solid, the band gap characteristics of the structure are further improved. The higher-order characteristic frequency rises, and therefore, the frequency corresponding to the band gap formed between the higher-order characteristic frequencies rises. However, it can be found that there is little difference in the initial frequency and the width of the first band gap between the two, indicating that the inner filling of the center node does not have much influence on these low-order characteristic frequencies.

In addition, as shown in Figure 5b, the band gap can be divided into upper and lower parts. It can be found that the initial frequency of the upper part of the band gap is not much different, but the width of the band gap gradually increases with the increase in the radius size of the central node. When the band gap reaches a certain size, the upper part of the band gap is divided into two parts, and the passband between the two bands gradually moves upward. The width of the first band gap in the upper part gradually increases, and the width of the second band gap gradually decreases. This is mainly due to the change in the structure, which makes the higher-order characteristic frequency increase, and the corresponding characteristic frequency in the passband increases continuously. In summary, the band gap properties of a solid structure are better than those of a hollow structure.

### 3.2. Band Gap Properties with the Different Ligament Width t of the Solid Ligament Structures

Through the analyses in Section 3.1, we found that the band gap of solid structures is more centered than that of hollow structures. In this study, the “centered band gap” describes that the band gap width is large, while the spacing between the band gaps is small, creating a more centralized appearance. Therefore, in this subsection, we investigate the effect of different ligament widths *t* of solid ligament structures on the band gap properties. For the four-ligament structures, as shown in Figure 3b, when the radius of the center node is 0.36 m, the width of the first band gap is 1505 Hz, which is an ideal width. Under this condition, the ligament width is changed to observe the change in its band gap. Also, considering the rationality of the structure, it is calculated here that the ligament width is increased from 0.02 m at an interval of 0.02 m to 0.18 m.

**Figure 6:**
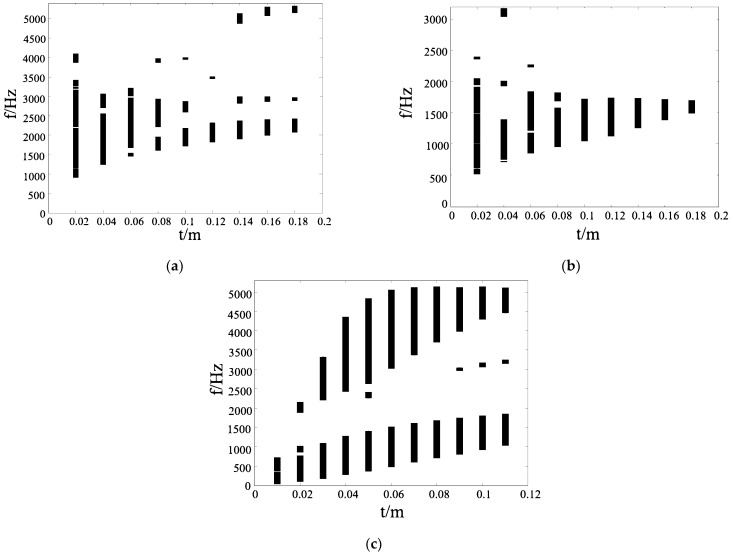
Band properties of the structures of different numbers of ligaments. (**a**) Four ligaments; (**b**) six ligaments; (**c**) three ligaments.

Figure 6 shows the band properties of the different ligaments. We investigate the characteristics of the band gap when the ligament width is increased from 0.02 m to 0.18 m in steps of 0.02 m. In Figure 6a, for the four-ligament structures, we can see that the smaller the ligament width, the wider the band gap, and the lower the onset frequency. The small passband range is due to the small difference in the values of certain order characteristic frequencies in the band structure diagram, which leads to the small difference between the cutoff frequency of the previous band gap and the starting frequency of the next band gap. After increasing to the certain size, the frequency width of the first band gap of the structure decreases and becomes undesirable. Moreover, the eigenfrequencies of all orders of the structure have increased, resulting in the band gap of the structure going upward. This is is mainly due to the increase in the ligament width, which makes the connection between the central nodes stronger and the structure has stronger stability.

For the six-ligament structure, as shown in Figure 4b, when *R* = 0.61 m, the second band gap of the structure is wider than that of other structures. By calculating the nearby values, it is found that when m is taken, the frequency range of the second band gap of the structure is 976–1759 Hz, the reduced frequency width is about 0.25, and the band gap is relatively ideal. Therefore, the radius of the central node of the structure is fixed to this value. Similar to the four-ligament structure, we calculated the band properties of the ligament band from 0.02 m to 0.18 m. In Figure 6b, the band gap of the six-ligament structure change pattern is similar to that of the four-ligament chiral structure. The smaller the ligament width, the wider the band gap width and the lower the onset frequency of the band gap; the greater the ligament width, the higher the stability of the structure, and the onset frequency of the band gap gradually increases. The number and width of the band gap also gradually become smaller.

As shown in Figure 5b, when the radius of the center node is 0.16 m, the width of the first band gap is largest. Now, we set the radius of the center node *R* = 0.16 m, and calculate the band gap under each structure when the ligament width is varied from 0.01 m and increased by 0.01 m step to 0.11 m. Figure 6c shows the band gap properties of the triligaments structure. With the increase in width *t*, the width of the first band gap first increases and then decreases, and the width of the first band gap reaches its maximum when the width of the ligament is nearly 0.05 m. The initial frequency of the band gap formed by the high-order characteristic frequency increases rapidly, and the cutoff frequency of the band gap increases first with the increase in the ligament width and basically stays unchanged after reaching a certain value; so, the band gap width increases first and then decreases. It can be found that when the ligament width is around 0.05 m, the band gap characteristics of the structure are the best.

The band gap characteristics of the triligaments chiral structure are better than those of other chiral structures. The band gap width is wider and more concentrated. The corresponding characteristic frequency variation in the passband of other chiral structures is more complex, which makes the band gap dispersed and the width not very ideal.

### 3.3. The Band Gap Properties of Symmetric Triligaments Structure

Through the analysis of bandgap structures in various ligament configurations in Section 3.1 and Section 3.2, we observed that the band gap properties of triligaments chiral structures are superior to those of other structures. Building upon this discovery, the impact of symmetry on the structural band gap was investigated. The rectangular lattice possesses two rotational symmetries, the tetragonal lattice has four rotational symmetries, and the triangular lattice exhibits six rotational symmetries. It was observed that under identical conditions, as the lattice symmetry increases, the crystal structure is more likely to exhibit an improved band gap [32]. This phenomenon has also been observed in previous studies on the influence of scatterers on the band gap. In this study, the chiral structure composed of three ligaments possesses rotational symmetry but lacks axial symmetry. The positional relationship between the ligaments and the central node in the geometric configuration has shifted from being tangent to being vertical.

The position of the central node of the vertical triligaments structure is similar to that of the triligaments chiral structure. Likewise, the size of the original cell size of the geometric structure remains unchanged. The ligament width is *t*, and the central node radius is *R*.

**Figure 7:**
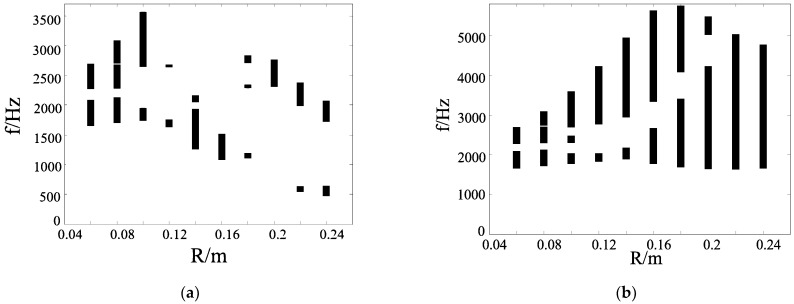
Band gap properties of vertical triligaments structure with radius of central node. (**a**) Hollow structure; (**b**) solid structure.

Figure 7 shows the band gap properties of the vertical triligaments structure with the different radius R of the center node. As shown in Figure 7a, with the increase in the radius of the central node, the passband between the band gaps expands, the width of the first band gap narrows, and the width of the other band gap increases. When the radius reaches around 0.1 m, the width of the second band gap is wider and the band gap is more ideal. However, with the increase in the radius of the central node, the band gap characteristics of the structure change greatly. It can be seen that when the radius is 0.12 m, the band gap width of the structure is narrower than that of the structure on both sides. When the radius of the central node is around 0.14 m, the band gap frequency is low and the width is good. As the radius size of the central node continues to increase, it can be found that no more ideal band gap appears.

As shown in Figure 7b, the structure has good band gap characteristics. With the increase in the radius of the central node, the band gap width of the structure increases gradually. When the radius of the central node is very small, the structure is equivalent to the hexagonal honeycomb structure, and the increase in the radius of the central node is equivalent to adding the concentrated mass at the corner of the hexagonal grid. From the variation in the band gap due to the size of the central node, it can be found that the band gap of the vertical triligaments structure is better than that of the regular hexagonal periodic grid structure. When the radius of the central node is less than 0.2 m, there is a passband between the band gap of the structure. With the increase in the radius of the central node, the corresponding frequency of this passband also increases gradually. The width of the band gap at the top of the passband first increases and then decreases, and the band gap at the bottom also increases slowly. It can be seen that when the radius of the central node is around 0.22 m, the band gap at the top has disappeared, and there is only one band gap in the structure, and this band gap is the widest. The corresponding frequency range is 1616–5028 Hz.

**Figure 8:**
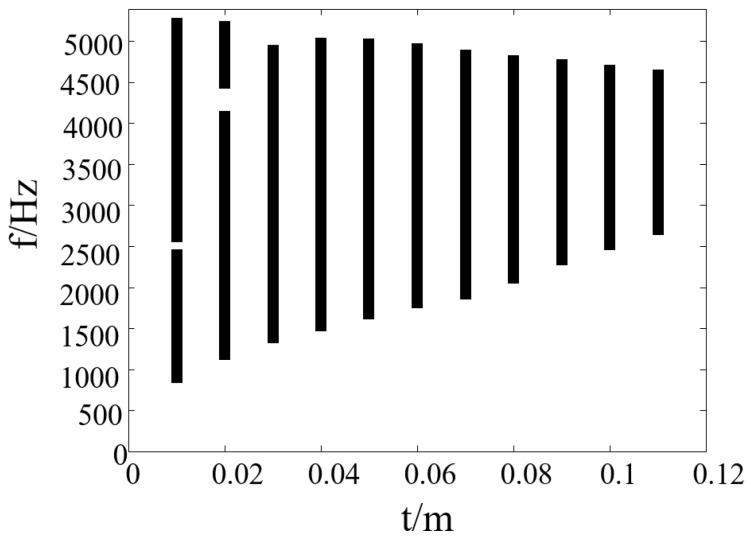
Band gap properties of vertical triligaments structure with ligament width.

Figure 8 shows the band gap properties of the vertical triligaments structure with the ligament width. When the ligament width is smaller, the band gap of the structure becomes wider. By increasing the ligament width of the structure, the starting frequency of the band gap gradually rises, while the width decreases gradually. However, it is worth noting that when there are small changes within a limited range, the variation in the band gap of the structure is not significant, indicating that the band gap of the structure exhibits good stability. It can be observed that even when the ligament width increases to 0.11 m, the width remains very ideal.

According to the numerical results of the band gap properties with different ligament widths, for hollow chiral structures, when the width of the ligaments is changed, the variation in the structural band gap is different. The lower the width of the ligaments of the four-ligament and six-ligament chiral structures, the larger the band gap range of the structures. The pattern of the chiral structures of three ligaments is the opposite. For solid chiral structures, the general rule is that the smaller the ligament width, the wider the band gap. Therefore, in this structure, when other conditions remain unchanged, the narrower the ligament width is, the more conducive it is to open the band gap.

## 4. Transmission and Vibration Characteristics of Periodic Honeycomb Structure

### 4.1. Transmission Characteristics

**Figure 9:**
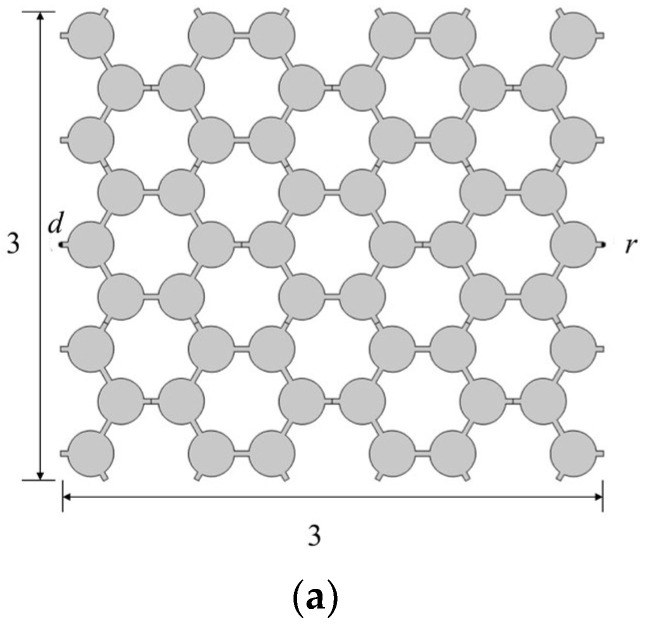

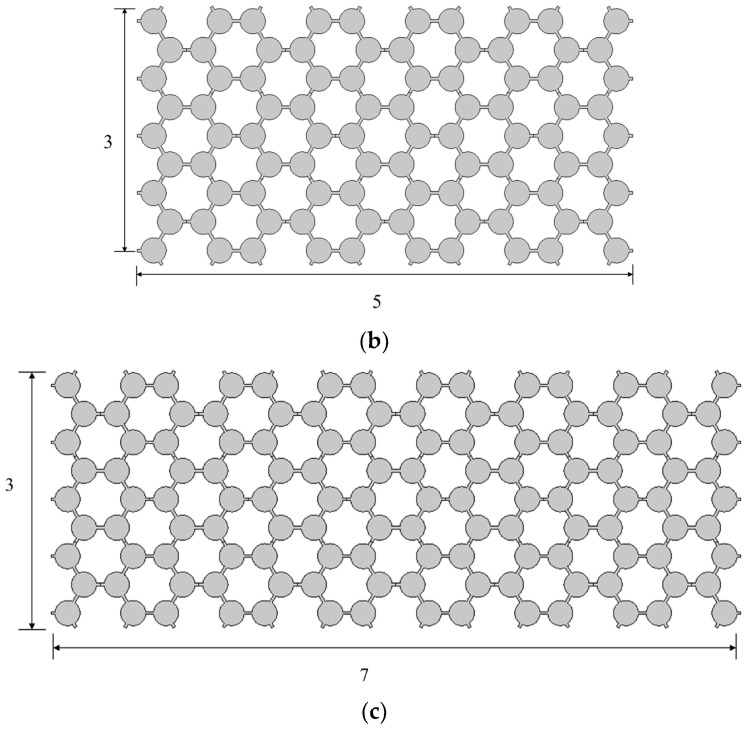
Finite periodic model of symmetrical triligaments structure. (**a**) 3 × 3; (**b**) 5 × 3; (**c**) 7 × 3.

In this section, we study the attenuation effect of different period numbers on elastic waves. The transmission equation can be expressed by $A_{f}=20\log\left(U_{r}/U_{d}\right)$, where $U_{d}$ is the excitation point displacement amplitude, $U_{r}$ is the response point displacement, and $A_{f}$ is the sound reduction index. Figure 9 shows the 3 × 3, 5 × 3, and 7 × 3 vertical triligaments period structure, respectively. The 3 × 3 here refers to the 3 horizontal and 3 column periodic structure. The 5 × 3 and 7 × 3 are similar. Unlike ligaments with chiral structures that are tangential to a circle, vertically structured ligaments refer to ligaments that are vertical to the corresponding tangential line. Letters *d* and *r* denote the excitation source and the receiver, respectively. The boundary condition is the free boundary. The direction of excitation and response is transversal.

**Figure 10:**
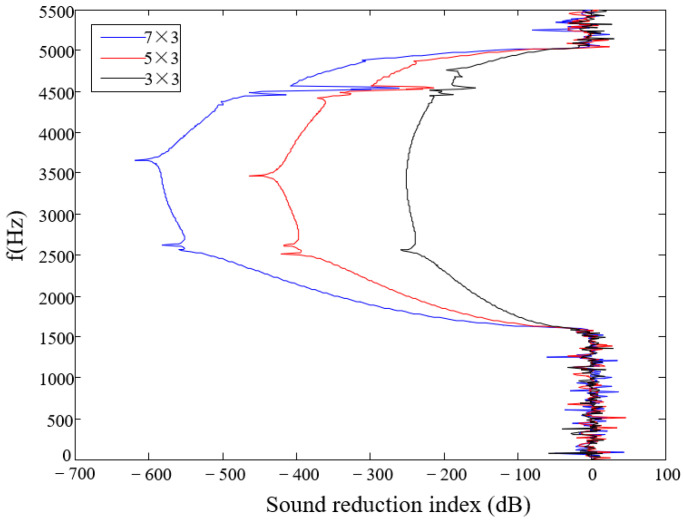
Frequency–response curves of symmetrical triligaments structure with different cycle numbers.

As can be seen from Figure 10, with the increase in the number of periods, the attenuation of the elastic wave increases. When there are only three cycles in the direction of the wave propagation, the maximum attenuation is still 200 dB. This demonstrates the excellent sound insulation properties of the structure.

### 4.2. Vibration Mode Characteristics

In this section, the relationship between the vibration mode and the vertical triligaments structure is studied. Therefore, we analyze the vibration modal patterns corresponding to the characteristic frequencies near the two wide band gaps and the vertex of the irreducible Brillouin region.

**Figure 11:**
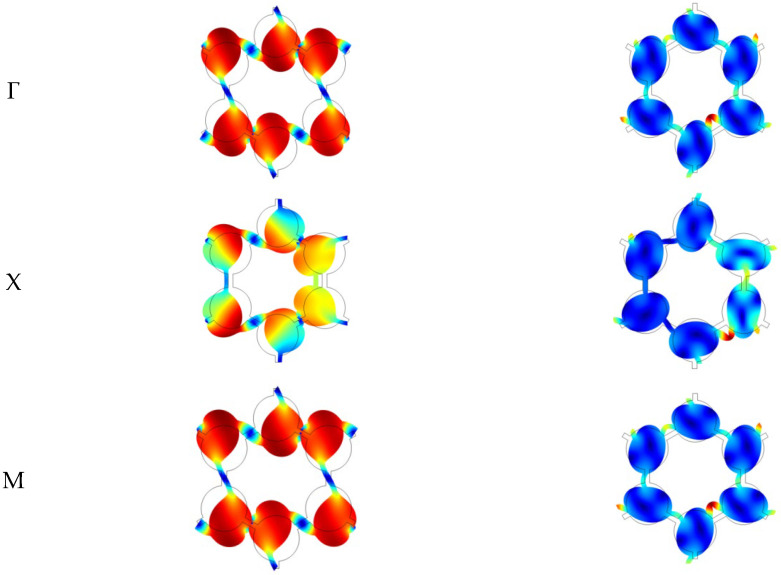
Unit cell mode of vertical triligaments structure.

By analyzing the band structure of the vertical triligaments structure, it is found that the structure’s band gap is defined by the 18th and 19th-order characteristic frequencies. The vibration mode of the vertical triligaments structure is illustrated in Figure 11. The redder the color of the cloud image, the larger the displacement, and the bluer the color, the smaller the displacement. It refers to the vibration mode corresponding to the vertex of the structure in the irreducible Brillouin region. Γ, X, and M represent the different vertexes of the irreducible Brillouin region, respectively. The relevant theory of the irreducible Brillouin zone can be found in [31]. The vibration modes at points Γ and M exhibit identical behavior. During the 18th-order characteristic frequency, the upper and lower nodes converge toward the center due to a modification in ligament connection, resulting in a reorganization of nodes into a two-row, three-column structure. This configuration introduces symmetry, with rotational symmetry observed in the mode shapes at points Γ and M, while the mode shapes at point X display axisymmetry.

Through observation of the band structure diagram, it is evident that the band gap is closely related to the characteristic frequencies corresponding to the irreducible Brillouin zone vertices. The vibrational modes of the structure at these positions play a significant role in determining the band gap. In chiral structures, under the same conditions, the number of ligaments have a considerable impact on the stiffness of the structure. A higher number of ligaments lead to increased structural stiffness. However, calculations of the band structure reveal that an increase in the number of ligaments does not necessarily result in improved band gap characteristics. In chiral structures with a triangular lattice unit cell, the one with three ligaments exhibits better band gap characteristics compared to the structure with six ligaments. By altering the connection between the ligaments and the central node from tangent to vertical, the symmetry of the unit cell structure is enhanced.

## 5. Harmonic Response Analysis of Periodic Structures

**Figure 12:**
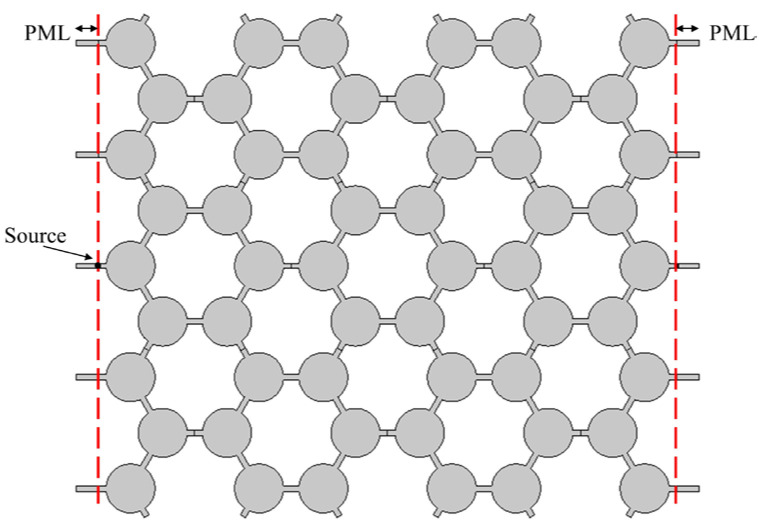
Harmonic response analysis model.

**Figure 13:**
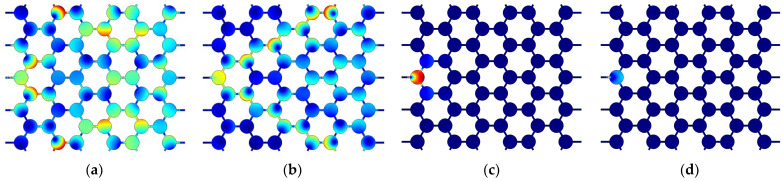
Displacement responses at different frequencies. (**a**) 500 Hz; (**b**) 1000 Hz; (**c**) 2000 Hz; (**d**) 3000 Hz.

The harmonic response analysis aims to evaluate how a structure responds to a simple harmonic load with known frequencies. In this investigation, simple harmonic loads are applied at frequencies corresponding to the passband and band gap of the structure to examine the displacement response and validate the band gap calculation. Figure 12 shows the harmonic response model. There are Perfect Matched Layer (PML) boundaries on the left and right sides of the model, which indicates that the wave is infinite in the transverse propagation region. The upper and lower boundaries are free boundaries. Similar to the chiral triligaments structure, three propagation cycles are computed in the propagation direction. It has been established previously that the band gap frequency range of the symmetric triligaments structure spans from 1616 Hz to 5028 Hz. Consequently, two frequencies from the passband (500 Hz and 1000 Hz) and two frequencies from the band gap (2000 Hz and 3500 Hz) are specifically chosen for analysis. The resulting displacement response is illustrated in Figure 13 below. Similar to Figure 11, the redder the color of the cloud image, the larger the displacement, and the bluer the color, the smaller the displacement.

In Figure 13, it is evident that when the frequency of the simple harmonic load aligns with a frequency within the band gap range, vibration is significantly dampened within a unit cell, showcasing the structure’s effective vibration reduction capabilities. Conversely, within the pass band, waves can propagate along the structure toward the right boundary. Notably, the wave propagation within the structure exhibits a clear sense of directionality. At a frequency of 1000 Hz, the angle between the direction of wave propagation within the structure and the x-axis of the model is approximately 30°, highlighting the directional nature of wave propagation in the analyzed system.

Due to the error of artificial processing or the consumption of use, the size of the structure is not strictly equal to the original size. The vibration isolation capacity of the structure should also stand the test in this case. In this study, we change the size of the solid nodes of the structure only. Since the size of the parts conforms to the normal distribution, 10% of the standard size is selected as variance. When the maximum wide band gap is generated by the symmetric triligaments structure, the structural parameters are a node radius of 0.22 m and a ligament width of 0.05 m. Random normal numbers with a mean value of 22 and a variance of 0.22 are generated. We select a set of data for simulation.

**Figure 14:**
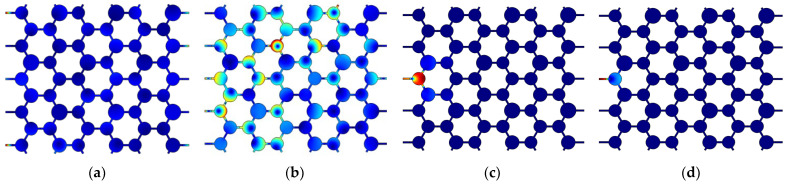
Displacement responses of different frequencies in different radius nodes. (**a**) 500 Hz; (**b**) 1000 Hz; (**c**) 2000 Hz; (**d**) 3000 Hz.

Similar to the original symmetric triligaments structure, the harmonic response displacement diagrams for 500 Hz, 1000 Hz, 2000 Hz, and 3000 Hz can be observed in Figure 14. The meaning of cloud image colors is similar to Figure 13. Despite the non-uniformity in the node sizes of the structure, it exhibits a notable vibration isolation effect, particularly within the specified frequency band gap. The vibration isolation performance remains robust across the mentioned frequency range. Consistent with the symmetric structure, the harmonic response analysis is conducted over a period of 3 × 3 cycles. The locations of the excitation points and response points remain unchanged from the previous structure, ensuring consistency in the analysis approach.

## 6. Calculation of Transient Fluctuations in Periodic Structures

In engineering, vibration frequency is the superposition of transform and multi-frequency vibration. In a previous harmonic response analysis, we simulated the response at a single frequency, and it was independent of time. In this subsection, we simulate the transient response with time, and the cosine envelope function is a common form of acoustic source used in engineering. This situation is more suitable for practical engineering applications. Analyzing transient waves in acoustic fields can enhance our comprehension of the propagation features of acoustic waves in a given space. By examining the transient waves within the acoustic field, we can gain insights into the propagation path and loss of sound waves, enabling a more comprehensive understanding of the design structure. Therefore, it is required that the designed phononic crystals and artificial periodic structures have a wide enough frequency range. Therefore, in order to check the band gap of the structure and observe the propagation changes in the wave within the structure, a transient wave calculation is carried out here. We add a pressure source to the center of the model. The wave can spread uniformly in all directions. The module of the acoustic–solid interaction physical field in COMSOL is selected and the transient study is added. The acoustic source has a center frequency of 3.5 kHz and a pulse duration of 2 ms [36]. The form of the acoustic source is shown in Equation (1). We can see that the cosine envelope function consists of the cosine function and the bichromatic/biharmonic expression [37,38]. Thus, the cosine envelope function contains bichromatic/biharmonic expression.

(1) $$f\left(t\right)=\left\{\begin{array}{c} 2\left[1+\cos\left(\frac{2\pi }{T_{c}}\left(t-\frac{T_{c}}{2}\right)\right)\right]\cos\left(2\pi f\left(t-\frac{T_{c}}{2}\right)\right)\\ 0,\;\;\;t<0,t>T_{c} \end{array}\right.,\;\;\;0\leq t\leq T_{c}$$

**Figure 15:**
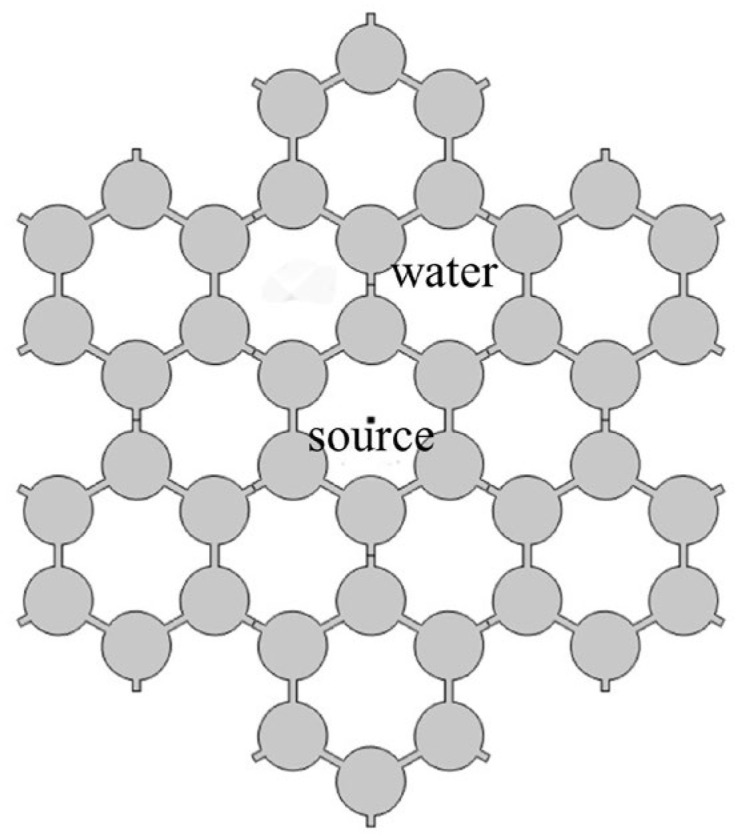
Transient fluctuation model of vertical structure of three ligaments.

**Figure 16:**
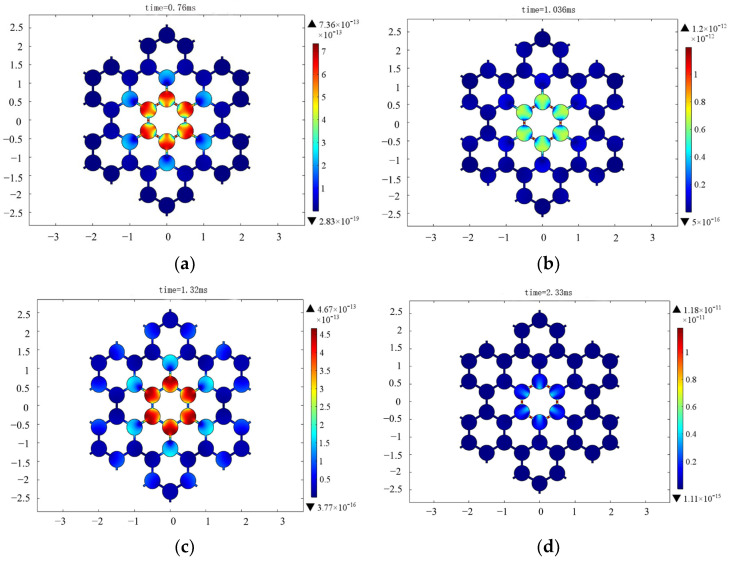
Displacement cloud diagram of solid symmetric triligaments structure at different times. (**a**) 0.76 ms; (**b**) 1.036 ms; (**c**) 1.32 ms; (**d**) 2.3 ms.

The calculation model of the transient fluctuation in the periodic structure is shown in Figure 15. The blank space between the structures is water. The sound source excites in the water and the sound waves travel all around. Figure 16 shows a displacement cloud diagram of a solid symmetric triligaments structure at different times. In order to observe the propagation in the structure, the transient fluctuation calculation is carried out. We observe the displacement of the structure in chronological order. The maximum response amplitude is selected at different times. By observing the cloud diagram, along the wave propagation direction, the displacement response of adjacent central nodes differs greatly, and the propagation of fluctuations is effectively suppressed by the structure. It can be seen that the multi-frequency vibration of the substructure within the band gap frequency range has an inhibitory effect.

## 7. Conclusions

Honeycomb structures have a wide range of applications in the field of vibration isolation and noise reduction. In this study, we designed a periodic structure that can isolate noise within the frequency range from 1500 to 5000 Hz. The size of the unit cell in the geometric structure was set to remain fixed, while the radius of the central node in the unit cell was varied. With a given central node, there was a ratio of the length of the ligaments to the distance between adjacent nodes’ centers, corresponding to a specific geometric configuration. The band gap characteristics of various chiral structures were then compared.

The meaningful conclusions are as follows: The triligaments structure has better band gap characteristics compared to other chiral structures. When the central node is hollow inside, the triligaments chiral structure exhibits good band gap characteristics under different structural configurations, whereas the band gaps of other chiral structures are dispersed and their widths are not ideal. Therefore, the chiral honeycomb structure with a triligaments configuration can achieve better vibration damping effects. When the central node is solid, the band gap characteristics of each structure are significantly improved, effectively increasing the width of the band gaps of each structure. The band gap of the triligaments chiral structure remains the most ideal, with a large band gap width and a moderate number of passbands. The change in ligament width leads to different variations in the band gap of the structure. For the four-ligament chiral structure and the six-ligament chiral structure, as the ligaments width decreases, the range of the band gap of the structure increases; meanwhile, for the triligaments chiral structure, it is the opposite. For solid chiral structures, a general rule is that a smaller ligament width corresponds to a wider band gap. Thus, in this structure, keeping other conditions constant, a narrower ligament width is more favorable for opening the band gap. By simultaneously adding axial symmetry and filling the central node, the band gap characteristics of the vertical triligaments structure are further improved compared to the band gap characteristics of the triligaments chiral structure. The larger the frequency range of the band gap of a structure, the more favorable it is for attenuating vibrations. Through harmonic load and transient wave propagation calculations, it is found that the structure can effectively suppress the propagation of waves within the band gap range. Our study makes a meaningful contribution to the design and manufacturing of sound insulation materials in related fields in engineering. However, there are still some inadequacies in our work, such as not considering the material and dimensional defects in the actual design of the structure. We also need to consider the impact of complex boundary conditions on wave propagation. We will continue to enhance our work in the future.

## Data Availability

The raw data supporting the conclusions of this article will be made available by the authors on request.
